# Editorial: Endogenous Viral Elements—Links Between Autoimmunity and Cancer?

**DOI:** 10.3389/fmicb.2018.03171

**Published:** 2018-12-20

**Authors:** Martin S. Staege, Alexander Emmer

**Affiliations:** ^1^Department for Operative and Non-operative Pediatric and Adolescent Medicine, Martin Luther University Halle-Wittenberg, Halle, Germany; ^2^University Clinic and Outpatient Clinic for Neurology, Martin Luther University Halle-Wittenberg, Halle, Germany

**Keywords:** endogenous retroviruses, endogenous bornaviruses, envelope proteins, patient derived xenografts, multiple sclerosis, gene expression, solid tumors, hematopoietic neoplasia

The association between cancer and autoimmune diseases has been known for a long time (Benvenuto et al., 2017). With the exception of some virus-induced tumors, cancer antigens are synthesized based on information that is present in the normal genome of the patient. Therefore, these antigens are usually highly similar or even identical to self-antigens. This fact may explain why antibodies in cancer patients often have a similar epitope spectrum compared to antibodies in autoimmune patients. In this regard, one can consider para-neoplastic autoimmunity as simple cross-reactivity between tumor cells and normal cells. In the present era of checkpoint-inhibition therapy for cancer, this phenomenon is obviously of great clinical importance. On the other hand, patients with some autoimmune diseases have an increased risk of developing cancer (Cristaldi et al., 2011). Hematopoietic neoplasia may be a consequence of prolonged immune cell stimulation by non-clearable auto-antigens. The interpretation of solid tumors emerging in patients with autoimmune disorders is probably more complicated. In this case, the never-ending stimulation of the immune system may force the development of regulatory cells that accidentally suppress immunocompetent cells with anti-cancer activity.

Interestingly, a common feature of cancer and autoimmune diseases is the altered expression of endogenous viral elements (EVEs). EVEs can be classified based on the exogenous viruses that are their most likely nearest relatives. In mammals, endogenous retroviruses (ERVs) represent the largest EVE family, and comprise nearly 10% of the human genome. Based on sequence similarities, this family can further be subdivided into several clades. Most ERV loci are transcriptionally inactive, but transcription has been observed under varying pathological conditions. In particular, ERV-encoded envelope proteins are considered as pathogenetic factors (Grandi and Tramontano; Gröger and Cynis).

The presence of substantial number of EVEs in the genomes of virtually all higher eukaryotes strongly suggests a physiological function for such elements. Indeed, host organisms employ some of these elements for important tasks e.g., placenta development, regulation of gene expression, or defense against exogenous pathogens. The liaison between the host and EVEs can approach true symbiosis, as in the example of endogenous viruses in some parasitic wasps (Federici and Bigot, 2003).

This *Frontiers Research Topic* compiles current aspects of EVE research, paying particular attention to ERVs and bornaviruses—two EVE families that are important in the context of human diseases. Like ERVs, endogenous bornavirus-like elements (EBLs) have been found in several vertebrates including humans (Fujino et al., 2014). The presence of these elements in cellular DNA is surprising because bornaviruses have no reverse transcriptase and no known DNA stage in their life cycle. However, integration of bornavirus-derived DNA into host genomes during natural infection seems to be a widespread event that is mediated by transposable elements from the host cells (Horie et al., 2010). Interestingly, EBLs have been shown to inhibit replication of exogenous bornaviruses in ground squirrels pointing to a probable function of EBLs (Horie et al., 2013). On the other hand, alterations in EBLs may be involved in cancer formation (Honda).

The exact number and chromosomal locations of EVEs vary even between closely related species. For instance, ERV3 is only present in most but not all old-world monkeys (Bustamante-Rivera et al.). The species specificity of EVEs offers a charming explanation for why some human diseases (e.g., certain cancers like Ewing sarcoma or Hodgkin lymphoma) do not occur (to our knowledge) or only occasionally occur in the rest of the animal kingdom. ERV3 is one of the best-studied human EVEs, but its function in health and disease is not clearly understood (Bustamante-Rivera et al.).

For most EVE/cancer associations, it remains unclear whether the expression of these EVEs is only a consequence of deregulated gene expression in cancer cells, or whether EVE are causally involved in cancer development. Not all EVEs are over-expressed in cancer cells in comparison to normal cells. Moreover, a single EVE can behave differently in different tumor models. Tumor cells proliferate by bypassing the control mechanisms that otherwise allow cell division only when physiologically necessary. *In vitro*, quasi-physiological cell proliferation can be studied in lymphocytes, which can be activated by varying stimuli mimicking the recognition of cognate antigens. A comparison between such activated B lymphocytes and neoplastic B cells indicates that different sets of ERV elements are transcribed in these two cell types (Attig et al.). Obviously, proliferation alone is not sufficient to induce the expression of a complete set of ERV elements. Tumor cells and their normal counterparts have more differences than just the lack of proliferation control. Another feature of cancer cells is their altered differentiation capacity. Germ cell tumors may be an interesting model for studying the interaction between ERV and the cellular differentiation status (Mueller et al.). In this model, ERV expression levels were inversely correlated with differentiation status. Such correlations between differentiation capacity and ERV expression may also be responsible for the shortened progression-free survival of sarcoma patients with high ERV expression (Giebler et al.). Differentiation potential and self-renewal are the two hallmarks that define stem cells. In this regard, the impact of ERV expression on stemness in normal and malignant cells is remarkable, and has been studied in melanoma (Balestrieri et al.). The malignant phenotype of these cells seems to be at least partially dependent on ERV expression. Similar pro-oncogenic effects have been observed for other repetitive elements, e.g., L1. Knockdown of L1 in urothelial carcinoma cells decrease their proliferation (Vasudevan et al.).

Cells have developed restriction systems that inhibit the propagation of exogenous viruses as well as of EVEs. One important group of factors involved in this restriction process are the cytidine deaminases of the apolipoprotein B mRNA editing enzyme family (APOBEC). As a side effect, these enzymes can also cause mutations in the cellular genome. The increased activity of APOBEC members after EVE re-activation might therefore be a mechanism by which oncogenic mutations are generated, and this effect might be one factor explaining the association between EVEs and cancer. As a proof of principle, the association between L1 and APOBEC activity was investigated in urothelial carcinoma (Vasudevan et al.). In this model, L1 only showed a small impact on APOBEC activity. Whether other EVE elements might exert APOBEC-dependent mutagenic effects should be investigated in the future.

An interesting aspect of so-called patient-derived xenografts (PDXs) is their frequent contamination with murine ERVs (Bock et al.). Today, we observe an unintelligible hype over PDX models, and one can get the impression that PDXs represent an absolutely important innovation that drastically advances research. The scientific community could probably use a reminder that passaging of tumor cells in immunocompromised animals was invented several decades ago as the only way of maintaining these cells outside of the patient. With the development of efficient cell-culture techniques, the necessity of passaging in live animals has principally been overcome, and the re-invention of animal passaging under the new name “PDX” does not seem well-founded (at least in a large proportion of applications). It is well-known that animal passages alter the biological behavior of cells (Sanford et al., 1959). Which roles ERVs play in this process requires further elucidation.

Gene expression in cancer cells—as seen by researchers—is an end product of a long-lasting co-evolution of the cancer cell population and the host organism's counter-strike mechanisms. Therefore, active EVE transcription in cancer cells can also be a consequence of activated defense mechanisms. As mentioned above, APOBEC activation is one mechanism by which cells restrict exogenous virus infections. EVEs may be a reservoir of endogenous activators in cases where exogenous viruses fail to activate these defense mechanisms (Bannert et al.). Such “virus-mimicry” (Bannert et al.) could be considered as part of a “SOS response” (Bustamante-Rivera et al.) that is activated in cells if they cannot respond purposefully to a given condition. This lack of a coordinated cellular reaction is a typical feature not only of cancer but also of autoimmunity. In the autoimmune situation, the immune system cannot eliminate the antigen because this antigen is an integral part of the body. The attacked tissue, on the other hand, cannot power down the attack because the aggressor is also an integral part of the body.

If synthesized at the wrong time, EVE-derived proteins can be harmful for the host organisms. It seems that such proteins—especially ERV envelope proteins—can induce unwanted immune responses and tissue damage (Grandi and Tramontano; Gröger and Cynis). For instance, such effects have been observed for the human syncytin ERVW1. ERVW1 and other ERV envelope proteins are considered to be pathogenetic factors in autoimmune diseases including multiple sclerosis. Gene expression analyses in a neuroblastoma model of CoCl_2_-simulated hypoxia identified another ERV envelope locus, ERV-FRD1, as a possible candidate for cytopathic envelope proteins in the context of neuronal diseases (Brütting et al.).

In addition to EVEs *sensu stricto*, eukaryotic genomes contain high numbers of EVE-like elements that have no similarity to known exogenous viruses or that have lost most of their genetic information in the course of evolution. The distinction between these classes is often blurred and sometimes a matter of opinion. ERVs are usually classified as repetitive elements, while the low copy numbers of other EVE classes prevented their inclusion in the corresponding databases. Taking into account the large number of different EVEs (ERVs) and related elements in eukaryotic genomes, the comprehensive analysis of these elements is not easy. The co-expression of EVEs and neighboring genes (Mueller et al.) may offer a new approach for the characterization of disease-associated EVE loci (Kruse et al.; Brütting et al.). Genome coordinates from gene expression experiments or other sources can be mapped to EVE coordinates in order to predict transcriptionally active loci. Using a new web tool that implements this approach, a new transcription start site was identified in the Hodgkin lymphoma-associated cytochrome 4 family Z member 1 gene (Kruse et al.).

Taken together, several EVEs are integral parts of the genomes of virtually all eukaryotic species. Clarifying the physiological and pathophysiological functions of these elements, as well as investigating the mechanisms that lead to altered EVE expression in disease contexts, may identify new targets for the treatment of conditions like cancer or autoimmune diseases.

## Author Contributions

All authors listed have made a substantial, direct and intellectual contribution to the work, and approved it for publication.

### Conflict of Interest Statement

The authors declare that the research was conducted in the absence of any commercial or financial relationships that could be construed as a potential conflict of interest.
